# A genotoxin associated with colorectal cancer linked to gut dysbiosis in children with cystic fibrosis

**DOI:** 10.1128/jb.00190-26

**Published:** 2026-06-09

**Authors:** Kaitlyn E. Barrack, Sarvesh V. Surve, Ana V. de Sousa Bezerra, Caitlin E. Murphy, Shannon M. Soucy, Miguel A. Aguilar Ramos, Rebecca A. Valls, Rebekah D. Ruff, Emily P. Balskus, Julie L. Sanville, Juliette C. Madan, George A. O’Toole

**Affiliations:** 1 Department of Microbiology and Immunology, Geisel School of Medicine at Dartmouth 12285 , Hanover, New Hampshire, USA; 2 Department of Biomedical Data Science, Geisel School of Medicine at Dartmouth 12285 , Hanover, New Hampshire, USA; 3 Department of Chemistry and Chemical Biology, Harvard University 1812, Cambridge, Massachusetts, USA; 4 Howard Hughes Medical Institute, Harvard University 1812, Cambridge, Massachusetts, USA; 5 Division of Pediatric Gastroenterology, Department of Pediatrics, Dartmouth Hitchcock Medical Center 22916, Lebanon, New Hampshire, USA; 6 Department of Psychiatry and Pediatrics, Dartmouth Hitchcock Medical Center 22916, Lebanon, New Hampshire, USA; 7 Geisel School of Medicine at Dartmouth 12285 , Hanover, New Hampshire, USA; University of Virginia School of Medicine 12349, Charlottesville, Virginia, USA

**Keywords:** cystic fibrosis, gut microbiome, colibactin, dysbiosis, physiology

## Abstract

Cystic fibrosis (CF) alters the gastrointestinal microbiome from an early age, leading to significant changes in microbial composition and functionality. This study explores the physiological and microbiological factors contributing to dysbiosis in children with cystic fibrosis (cwCF), characterized by an increase in potentially pathogenic *Escherichia coli* and a decrease in beneficial anaerobes, such as *Bacteroides*. We employed an *in vitro* medium representative of the nutritional environment of the CF colon to test the role of factors, including mucin, fat, bile, pH, antibiotics, and features associated with inflammation (e.g., nitrate, sulfate, formate, and reactive oxygen species), on the growth of clinical isolates of *E. coli* and *Bacteroides* spp., with a focus on *Bacteroides vulgatus*. We further examined interactions between these two microbes under CF-like conditions and identified glycerol, a surrogate of increased fat, as a significant driver of altered microbial competition. Finally, we investigated genetic determinants influencing these microbial interactions by performing a transposon mutagenesis screen in *E. coli*. The results pointed to the role of colibactin, a DNA-damaging genotoxin associated with an increased risk of colorectal cancer (CRC), in mediating this microbial competition. This work enhances our understanding of the mechanisms of microbial competition in the CF gut and potential CRC risk in persons with CF through the identification of early-life microbial biomarkers.

**IMPORTANCE:**

The risk of CRC development in CF populations is significantly increased. This *in vitro* study examines the interplay of altered intestinal physiology in the microbial dysbiosis common in the CF gut, implicating the high-fat/glycerol environment in a competition-mediated depletion of immune-modulating *Bacteroides vulgatus*. This work identifies candidate features of the young CF intestine and gut microbiome that may contribute to dysbiosis, development of inflammation, and CRC in these populations, informing potential prognostic and therapeutic approaches.

## INTRODUCTION

The cystic fibrosis (CF) gastrointestinal (GI) tract is altered both physiologically and microbiologically from a young age (1–6). Key taxonomical differences observed in CF stools include increased Proteobacteria, largely driven by *Escherichia coli* (3–7), and decreased anaerobes that produce short-chain fatty acids (SCFAs), such as *Bacteroides, Akkermansia, Faecalibacterium,* and *Roseburia* (3, 6, 8). These differences are reported as early as 6 weeks of age in children with CF (cwCF) (1). Many factors likely contribute to this microbial imbalance, with large contributions from altered physiology (9), including nutrition (10–12), antibiotic use (13), and microbial competition. However, the role of such factors has not been thoroughly investigated in the context of CF.

Previous research investigated the role of increased fat in the CF intestine, driven by diet and fat malabsorption (14, 15), on the enrichment of *E. coli* in stool (7). *E. coli* isolates originating from a CF donor were better adapted to growth conditions supplemented with glycerol, a surrogate for increased fat, than isolates originating from a non-CF donor (7). CF isolates not only grew to a greater extent on glycerol but also exhibited a decreased stress response to glycerol as a sole carbon source compared to non-CF isolates. Instead of upregulating glycerol-utilization genes, which would be expected, CF isolates appear to lose the growth inhibition and stress response signals typically induced by glycerol (7). These findings implicate the role of fat and its breakdown products in shifting microbial abundance and function, while driving adaptation to altered physiology in the context of CF.

A dysbiotic intestinal environment and microbial composition modulate functionality in CF (16, 17), delay gut microbiome maturation (3, 6), and contribute to increased inflammation (18). From early ages, cwCF have increased fecal markers of inflammation (17, 19), which correlate with the microbial markers of dysbiosis, namely increased *E. coli* (19). Importantly, gut dysbiosis and inflammation are implicated in the development of colorectal cancer (CRC) in the general population; persons with CF (pwCF) have 10 times the risk of developing CRC compared to the general population (20, 21). Mechanisms underlying this increased risk in CF remain undetermined. In non-CF populations, pathogenic *E. coli* strains capable of producing the genotoxin colibactin, which promotes DNA damage, via expression of a polyketide synthesis (*pks*) island have been linked with increased CRC risk, microbial dysbiosis, and inflammation (22–25). However, limited research has investigated the role of colibactin in CF populations, and there is inconclusive evidence of increased prevalence of *E. coli pks+* strains for pwCF (26, 27). Thus, determining physiological and/or microbiological biomarkers, especially in early life, is imperative to understanding the mechanisms of CRC development and improving therapeutic potential in CF.

In this study, we aimed to identify physiological features of the CF intestine that may be involved in driving microbial dysbiosis, namely increased *E. coli* and decreased *Bacteroides*, using a previously developed *in vitro* medium that models the CF gut (28). We investigated the dynamics of the microbial interaction under various CF-like physiologies, highlighting an important role for glycerol in this intraspecies interaction. Finally, we probed the genetic components of *E. coli* involved in modulating the interaction with *Bacteroides*, identified genes involved in glycerol metabolism and colibactin synthesis, and characterized the prevalence and function of the colibactin-encoding *pks* island in CF and non-CF *E. coli* isolates and stool metagenomes. Our results suggest increased *pks* gene prevalence and abundance in CF stool metagenomes, along with trending increases in colibactin production in CF stool. Our work identifies microbial and physiological factors in young cwCF potentially contributing to the risk of developing inflammation and CRC later in life.

## RESULTS

### *Bacteroides vulgatus* is sensitive to CF-relevant physiological features and to *E. coli* competition

To test the hypothesis that physiological features relevant to the CF intestine may be driving taxonomical differences, we decided to focus on two key microbes that are consistently and significantly differentially abundant in CF cohorts compared to non-CF cohorts: *E. coli* and *Bacteroides* species (spp.) (1, 4–6, 17, 19, 29–32). Metagenomic studies have specifically identified *B. vulgatus* (recently renamed *Phocaeicola vulgatus* but referred to as *B. vulgatus* in this study) as depleted in CF stool samples (6, 19). One children’s study reported a significant depletion of *B. vulgatus,* but not *B. fragilis* or *B. thetaiotaomicron,* in CF children’s stool compared to those without CF (19). To date, a mechanism for this depletion has not been identified. To address this gap, we grew CF and non-CF clinical isolates of *B. fragilis, B. thetaiotaomicron, B. vulgatus,* and *E. coli* (Table S1) in CF-MiPro (28), a recently developed CF intestinal medium designed and validated to mimic the CF colon nutritional environment. CF-MiPro includes excess fats, mucin, bile, reactive oxygen species (ROS), alternative nutrients associated with inflammation (e.g., nitrate, sulfate, and formate), lower pH, and sub-lethal antibiotic levels. CF-MiPro was developed using MiPro, a medium mimicking the healthy gut environment, as the medium base (33); then, the aforementioned features were supplemented at a gradient of concentrations to represent low and median levels reported in CF stool samples (28).

**Fig 1:**
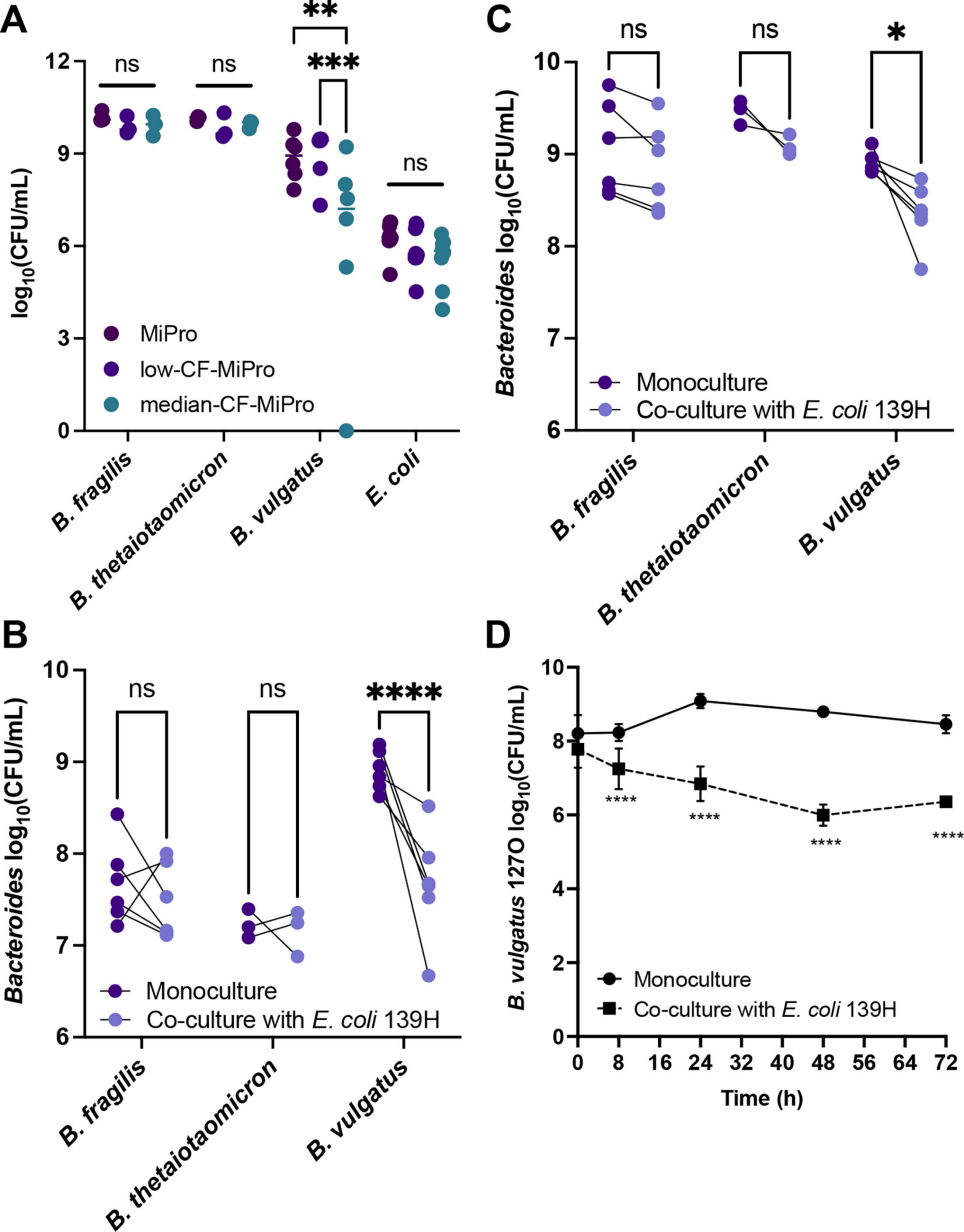
*B. vulgatus* is susceptible to CF-like growth conditions and competition with *E. coli.* (**A**) Clinical isolates of *B. fragilis* (*n* = 3), *B. thetaiotaomicron* (*n* = 3), *B. vulgatus* (*n* = 5), and *E. coli* (*n* = 6) were grown anaerobically in monoculture in MiPro (dark purple), low-CF-MiPro (purple, wherein CF-relevant features are spiked into MiPro at low concentrations), and median-CF-MiPro (teal, wherein CF-relevant features are spiked in MiPro at median concentrations) for 24 h. Growth is quantified via log_10_(CFU/mL). (**B and C**) Clinical isolates of *B. fragilis* (*n* = 6), *B. thetaiotaomicron* (*n* = 3), and *B. vulgatus* (*n* = 5) were co-cultured anaerobically with a CF clinical isolate of *E. coli*, 139H, in a 1:1 ratio in (**B**) BHIS pH 7 or (**C**) MiPro pH 7 for 72 h. Growth of each respective *Bacteroides* strain is displayed as log_10_(CFU/mL) on the y-axis when in monoculture (dark purple) or co-culture with *E. coli* (light purple), with a line connecting each strain. (**D**) Growth kinetics, as measured by CFU/mL, of one *B. vulgatus* strain, 127O, in co-culture with *E. coli* 139H in MiPro pH 7 over the course of 72 h. Statistical analysis performed using two-way ANOVA with Šídák’s multiple comparisons (*, *P* < 0.05; **, *P* < 0.01; ***, *P* < 0.005; ****, *P* < 0.001). Error bars represent mean +/− SEM.

We hypothesized that clinical isolates of *E. coli* would have a growth enhancement in CF-MiPro, while the viability of *B. vulgatus* clinical isolates grown in monoculture would be reduced in this *in vitro* environment, mirroring what is seen in the CF gut microbiome samples (19). Indeed, after 24 h of growth in MiPro, low-CF-MiPro, and median-CF-MiPro, there is an observed dose-dependent depletion of all tested *B. vulgatus* clinical isolates (Fig. 1A). This depletion is neither observed in the tested *B. fragilis* nor *B. thetaiotaomicron* strains, suggesting increased tolerance to CF-MiPro and thus a CF-like intestinal environment for these species. *E. coli* clinical isolates do not exhibit a growth enhancement in CF-MiPro and show similar colony-forming units per milliliter (CFU/mL) across all conditions. We observed a trend toward reduced viability in median-CF-MiPro, but this difference was not significant.

Next, we investigated the microbial competition between *E. coli* and *Bacteroides* species, as microscopic evidence via fluorescent *in situ* hybridization (FISH) shows their co-localization in inflammatory bowel disease (IBD) patient biopsies (34). Altered microbial interactions likely occur in the CF intestinal lumen due to the microbiological and nutritional environments (35), which may exacerbate the observed dysbiosis. Here, we co-cultured a CF isolate of *E. coli* (139H) in a 1:1 ratio with strains of *B. fragilis* (*n* = 3 strains)*, B. thetaiotaomicron* (*n* = 3 strains), and *B. vulgatus* (*n* = 6 strains) for 72 h in anoxic conditions. Using *Bacteroides* monoculture growth (assessed by CFU/mL) as a positive control, we found that *B. vulgatus* viability was significantly reduced in the presence of *E. coli* 139H in two separate rich media conditions buffered to pH 7: supplemented brain-heart infusion broth (BHIS) and MiPro (Fig. 1B and C). This reduction was not observed in *B. fragilis* or *B. thetaiotaomicron* co-cultures, although there was a trend toward reduced viability for both species. We confirmed that the reduction of *B. vulgatus* viability occurred independently of *E. coli* growth, as *E. coli* CFU/mL in co-culture was not significantly increased compared to its monoculture controls (Fig. S1A).

To observe the timing of *B. vulgatus* viability reduction by *E. coli*, we performed a growth kinetics assay with CF isolate *B. vulgatus* 127O and *E. coli* 139H in MiPro pH 7 for 72 h, collecting CFU/mL of each strain at 8, 24, 48, and 72 h. Reduction of 127O viability was observed as early as 8 h in co-culture with *E. coli*, compared to monoculture controls (Fig. 1D). This reduction in viability was largely independent of *E. coli* growth, whose viable counts were stable or slightly decreased at early time points in co-culture with *B. vulgatus* (Fig. S1B). These results suggest baseline competition between these two microbes under *in vitro* conditions that mimic the healthy intestine. Next, we explored how this interaction was mediated in CF-like conditions.

### Linear regression identifies bile and glycerol as key contributors to the reduction of *B. vulgatus* viability in the context of the CF intestinal environment

To investigate how CF-relevant physiological features impact intestinal bacterial growth and competition, we prepared MiPro medium with individual components altered to CF-MiPro concentrations. We grew clinical isolates of *B. vulgatus* (*n* = 6) and *E. coli* (*n* = 8) in mono- and co-culture for 72 h. Tested components included: MiPro pH 7 (control), MiPro pH 6, or MiPro pH 7 individually supplemented with mucin, bile salts (a 1:1 mix of cholic acid and deoxycholic acid), glycerol, sodium nitrate, sodium sulfate, sodium formate, hydrogen peroxide, or Bactrim, an antibiotic commonly used in CF clinics. See the Materials and Methods for the respective concentrations of each component. After 72 h, CFU/mL was calculated for each species, and metadata were assigned (Table S2). A linear model analyzed the contribution of each feature to the CFU/mL of each species.

Unsurprisingly, species and microbial competition (monoculture vs. co-culture) each played a significant role in overall CFU/mL (Table S3). Together, species (*P* < 0.001) and competition status (*P* = 0.007) contributed to 3.5% of the variance in the model. To account for these contributions and determine the physiological features impacting each species under different competition statuses, we subset the metadata by species and co-culture and then performed separate linear models for each category: *E. coli* in monoculture (Table S4), *E. coli* in co-culture (Table S5), *B. vulgatus* in monoculture (Table S6), and *B. vulgatus* in co-culture (Table S7).

*E. coli* survival in monoculture was significantly increased by numerous CF-like physiological features, including mucin (*P* = 0.003), bile (*P* = 0.001), glycerol (*P* = 0.002), nitrate (*P* = 0.003), sulfate (*P* = 0.007), and formate (*P* = 0.014) compared to the MiPro pH 7 control (Table S4). The sum of all tested features accounted for 17.8% of the variance in the model. Notably, the degree of response or change in CFU/mL of each feature (as represented through the “Estimate” parameter) was minimal (<1), indicating that while significant, the shifts in survival were modest across *E. coli* strains. In co-culture with *B. vulgatus,* bile (Estimate = 0.03, *P* = 0.049) and glycerol (Estimate = 0.06, *P* = 0.001) increased *E. coli* growth, while hydrogen peroxide (Estimate = −0.26, *P* =< 0.001), pH 6 (Estimate = −0.3, *P* < 0.001), and antibiotics at low-CF-MiPro (Estimate = −0.33, *P* < 0.001) and median-CF-MiPro concentrations (Estimate = −0.31, *P* < 0.001) decreased *E. coli* growth relative to the MiPro pH 7 control (Table S5). The sum of all tested features accounted for 26.6% of the variance in the model.

**Fig 2:**
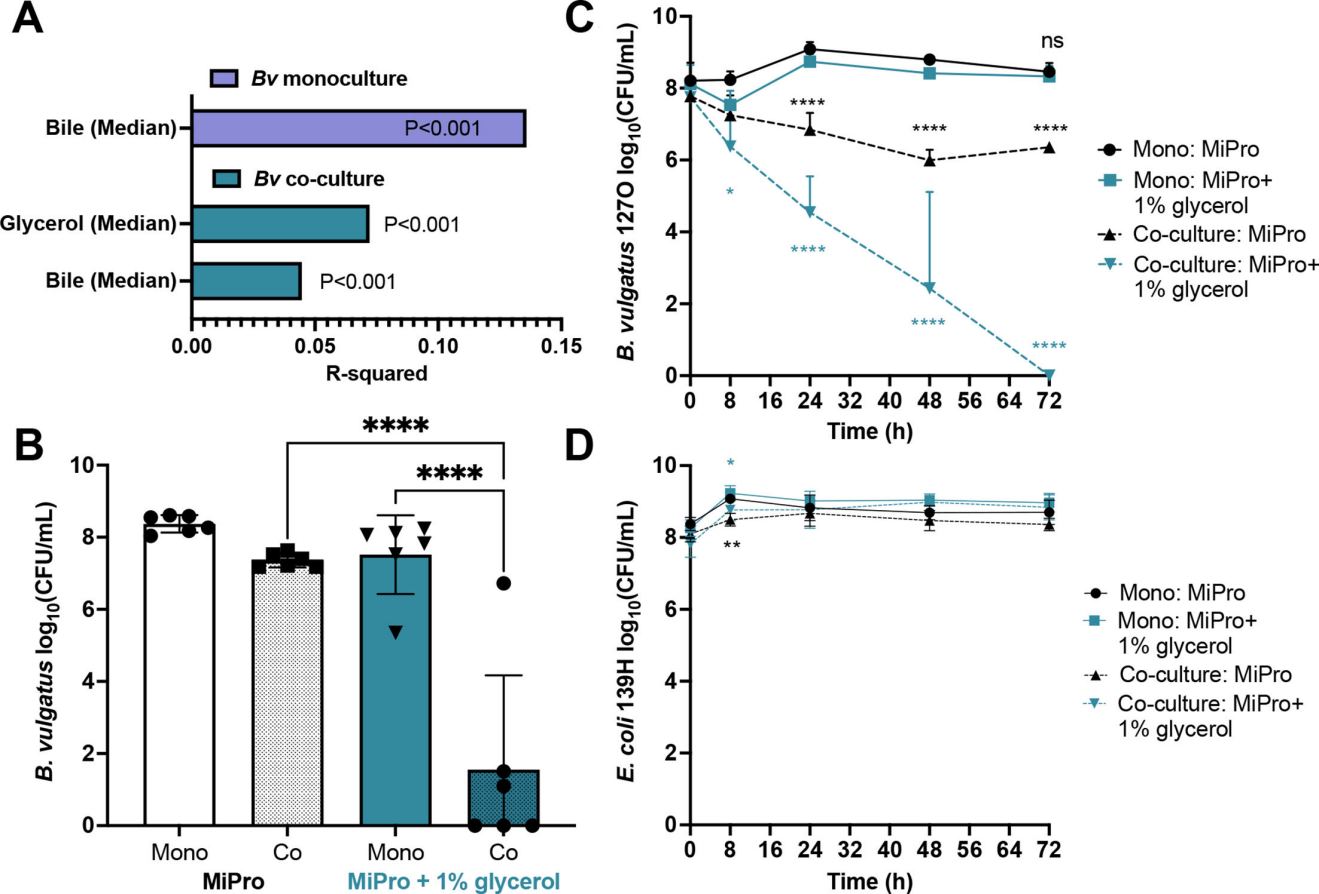
Linear regression identifies key components of CF gut physiology that contribute to *B. vulgatus* growth defects. (**A**) Significant features that contribute to the loss of viability of *B. vulgatus* (*“Bv,”* strain *n* = 6) in monoculture (purple) and in co-culture with *E. coli* (strain, *n* = 8; teal), compared to MiPro. “Median” denotes the concentration of each feature to be at median-CF-MiPro levels. R-squared values explain the contribution to variance in the model of each feature. (**B**) Endpoint survival of *Bv* strains in monoculture (“mono”) or co-culture with *E. coli* 139H (“co”) for 72 h growth in MiPro (black/white) or MiPro supplemented with 1% glycerol (teal). Each point denotes an individual *Bv* strain (*N* = 6 strains, average of 3 biological replicates). Statistical analysis performed using ordinary one-way ANOVA with Tukey’s multiple comparisons. (**C**) Growth kinetics of one *Bv* strain, 127O, in monoculture (solid lines) or co-culture with *E. coli* 139H (dashed lines) in MiPro (black) or MiPro + 1% glycerol (teal) over the course of 72 h. (**D**) Comparative *E. coli* 139H growth kinetics. Statistical analysis performed using two-way ANOVA with Šídák’s multiple comparisons between co-culture and monoculture CFU/mL within the same growth medium (*, *P* < 0.05; **, *P* < 0.005; ****, *P* < 0.001; ns, not significant). Error bars represent mean +/− SEM.

*B. vulgatus* survival in CF-like conditions was significantly impacted by fewer features, compared to *E. coli*. In monoculture, only bile at median concentrations had a significant negative impact on survival (Estimate = −5.17, *P* < 0.001, Fig. 2A; Table S6). The sum of the influences of all tested features accounted for 26.2% of the variation in the model. In co-culture with *E. coli*, bile (Estimate = −3.36, *P* < 0.001) and glycerol (Estimate = −3.15, *P* < 0.001) were negatively associated with *B. vulgatus* survival (Fig. 2A; Table S7). All tested features accounted for 14.4% of the variation in the co-culture model.

In all models, the tested features had relatively low contribution to overall variation (adjusted R^2^), ranging from 14.4% to 26.6%. This finding suggests that, individually, the CF-relevant features account for modest shifts in growth. However, in the context of the CF gut, these features are working in combination with each other, which likely compounds the observed growth defects. Thus, a more comprehensive approach is needed to properly address this question.

Bile’s negative impact on *B. vulgatus* survival was initially surprising, as *Bacteroides* species are generally considered bile-tolerant (36). Bile esculin agar, the selective medium used to isolate *Bacteroides fragilis*, contains high bile concentrations (37). These microbes possess enzymes that modify bile acid chemistry (e.g., deconjugation/conjugation with amino acids in the case of bile salt hydrolases [38]) to detoxify primary conjugated bile acids and promote colonization (39). However, bile acid modifications may lead to more toxic metabolites (40). One study reported an inhibitory effect of deoxycholic acid (DCA) on *Bacteroides* spp., including *B. vulgatus,* due to intracellular accumulation (41). The inhibitory effects of bile acids are strain-specific, aligning with our *B. vulgatus* monoculture growth data (Table S8). The impacts of bile on *B. vulgatus* persist in co-culture with *E. coli*, suggesting bile as a microbial stressor regardless of competition.

We decided to pursue the role of glycerol (a surrogate of fat) on modulating *B. vulgatus* growth for two reasons: (i) to our knowledge, glycerol has not yet been implicated in *Bacteroides* depletion in the context of CF, and (ii) glycerol only reduces *B. vulgatus* viability when *E. coli* is present, suggesting the role for microbial competition. First, we quantified free glycerol in stool from CF (*n* = 10) and non-CF (*n* = 12) children to reaffirm its appropriate use as a fat surrogate. Free glycerol, not incorporated into triacylglycerols (TAGs), is a direct measure of available glycerol in stool rather than what is hydrolyzed from TAGs in the processing of the fecal extracts for analysis. Free glycerol may accumulate in the CF intestine due to a lack of absorption after liberation from TAGs by pancreatic or microbial lipases; however, this point has not been explored. Using processing methods lacking saponification (e.g., strong base or acid) to avoid hydrolysis of glycerol from TAGs and ultra performance liquid chromatography/tandem mass spectrometry (UPLC–MS/MS), we successfully quantified free glycerol in stool samples from cwCF with healthy age-matched controls (Fig. S2). Non-CF stool samples averaged 0.01675 μg/mg free glycerol, and CF stool samples averaged 0.1515 μg/mg free glycerol (*P* = 7.4e−05), signifying an 8-fold increase in CF stool. Statistical analysis was performed using a mixed effect linear model to account for genotype, with age of donor and batch effects controlled for as the random variables.

Next, we investigated the dynamics of glycerol-mediated reduction of *B. vulgatus* viability in the presence of *E. coli*. At 72 h, *B. vulgatus* viability was significantly reduced across six clinical isolates in co-culture with *E. coli* when glycerol was supplemented in MiPro pH 7 (Fig. 2B). This depletion was observed in almost all strains of *B. vulgatus*, with 50% (*n* = 3 strains) exhibiting viability below detection. The low viability was independent of increased *E. coli* growth in glycerol (Fig. S3). We tracked the death curve of *B. vulgatus* strain 127O over the course of 72 h co-culture with *E. coli* 139H (Fig. 2C), observing 127O depletion as early as 8 h, with enhanced killing at 24 h compared to the MiPro pH 7 co-culture comparison. By 72 h, 127O CFU/mL were below detection, independent of *E. coli* 139H growth in glycerol (Fig. 2D).

Interestingly, this interaction was exclusive to live cell cultures; cell-free supernatants from *E. coli* 139H monocultures or co-cultures with *B. vulgatus* did not recapitulate the reduction of *B. vulgatus* viability (Fig. S4). We tested anoxic monoculture supernatants from (i) *E. coli* 139H grown in both MiPro pH 7 and MiPro pH 7 + 1% glycerol for 72 h and (ii) *E. coli* 139H and *B. vulgatus* CFPLTA003-2B in MiPro or MiPro + 1% glycerol for 72 h, exposing *B. vulgatus* strains (*n* = 5 strains) and *E. coli* 139H in monoculture to diluted fractions of the supernatants and quantifying growth via CFU/mL after 24 h (Fig. S4). None of the supernatant conditions inhibited *B. vulgatus* in monoculture, compared to the control conditions lacking supernatant. The only exception observed was the supernatant from *E. coli-B. vulgatus* co-culture in MiPro slightly reduced *B. vulgatus* 127O viability in monoculture, but not to the same degree as observed with live cells. These results suggest that the reduction of *B. vulgatus* viability in the presence of glycerol and *E. coli* requires cell-to-cell contact or involves an unstable or transiently acting secreted metabolite or toxin.

### Polyketide synthase (*Pks*)-encoded colibactin is involved in modulating the *E. coli-B. vulgatus* interaction in the presence of glycerol

To address the mechanism of *E. coli-B. vulgatus* competition, we performed a genetic screen to identify *E. coli* mutants that do not reduce *B. vulgatus* viability in co-culture with *E. coli* 139H when grown in MiPro + 1% glycerol. *E. coli* 139H was conjugated as a recipient with an *E. coli* strain donor containing a plasmid harboring the Tn*M* mariner transposon, equipped with a gentamicin resistance cassette. Nearly 10,000 colonies were generated in this mutant library, representing ~2× coverage of the 139H genome (Table S9).

Individual insertion mutants were screened for their ability to grow similarly to the *E. coli* 139H WT parent in monoculture and co-culture with *B. vulgatus* 127O in MiPro + 1% glycerol after 72 h anaerobic growth, without reducing the viability of *B. vulgatus* 127O growth in co-culture relative to its monoculture control. Candidates showing “full,” “partial,” or “minimal” restored viability relative to the *B. vulgatus* 127O monoculture controls were selected for secondary and tertiary screens. The secondary screen repeated the primary screen process, and the tertiary screen validated *E. coli* candidates in co-culture with five *B. vulgatus* isolates to confirm that the rescue in viability was not specific to 127O alone. A visual schematic of the transposon screen procedure is outlined in Fig. S5.

We obtained 549 primary candidates (5.6% of screened colonies), with a range of rescue capabilities: 87 (0.9%) of mutants restored *B. vulgatus* 127O viability fully relative to monoculture, 173 (1.8%) mutants restored viability partially, and 289 (2.9%) mutants restored viability minimally (Table S9). Only 51/549 candidates were validated in the secondary screen to restore *B. vulgatus* 127O viability fully. For the primary “full restoration” candidates (*n* = 87) that displayed only partial restoration of viability in the secondary screen, we denoted these as secondary “partial restoration” candidates, of which there were 24.

The location of transposon insertion in the candidate strains was determined via arbitrary primed PCR and Sanger sequencing of flanking regions, as described in the Materials and Methods. From this screen, we identified candidates that were categorized into genes related to metabolism, stress response, biofilm formation and its regulation, phage, rRNA, and pathogenicity (Tables S10 and S11). Of note, one full restoration candidate (*dhaR*::Tn*M*) and three partial restoration candidates (*dhaK*::Tn*M, dhaL*::Tn*M*, and *gldA*::Tn*M*) related to glycerol metabolism, serving as positive controls confirming the screen’s validity and the potential role of glycerol metabolic by-products (i.e., methylglyoxal) as toxic mediators in the interspecies interaction. Other metabolic genes with transposon insertions included *actP*, an acetate symporter, *malE*, a maltose-binding protein, and *alsK*, an allose kinase. These candidates likely suggest the importance of nutritional competition of these substrates in microbial interaction. Other mutants with full restoration phenotypes involved stress responses to oxidative stress (*sodA*::Tn*M*) or misfolded proteins (a *clpQY* promoter::Tn*M*), and biofilm maintenance (*pdeH*::Tn*M*) or regulation (*rsmE*::Tn*M*), suggesting mechanisms of competition involving stress tolerance through detoxification or biofilm formation.

Strikingly, 51% of the validated “full restoration” candidates (26/51) involved colibactin biosynthesis, a genotoxin associated with increased colorectal cancer risk (42–46). The *pks,* or polyketide synthase, gene cluster is a 54 kb genetic island with 19 *clb* genes encoding various non-ribosomal peptide synthetase (NRPS), polyketide synthetase (PKS), hybrid NRPS-PKS, and other biosynthetic enzymes, as well as accessory proteins (25, 44). Colibactin production is largely regulated by the first two genes in the cluster, *clbA* and *clbR,* and sets of genes in the cluster are transcribed independently. Specifically, there are four polycistronic elements (1: *clbA, clbR,* 2: *clbCDEFG*, 3: *clbIJKLMN*, and 4: *clbOPQ*) and three monocistronic elements (*clbB*, *clbH*, and *clbS*). The biosynthetic scaffold coordinates production of the inactive biosynthetic intermediate precolibactin, which contains two *N-*myristoyl-d-Asn motifs. After export of precolibactin from the cytoplasm, the biosynthetic enzyme ClbP, a periplasmic transmembrane peptidase, cleaves the prodrug motifs, triggering production of the active colibactin molecule, characterized by two electrophilic cyclopropane warheads (47–49). These warheads alkylate DNA at adenine-rich motifs (5′-AAWWTT-3′, where W = A or T), causing DNA interstrand cross-linking and activation of DNA repair machinery (50–52).

**Fig 3:**
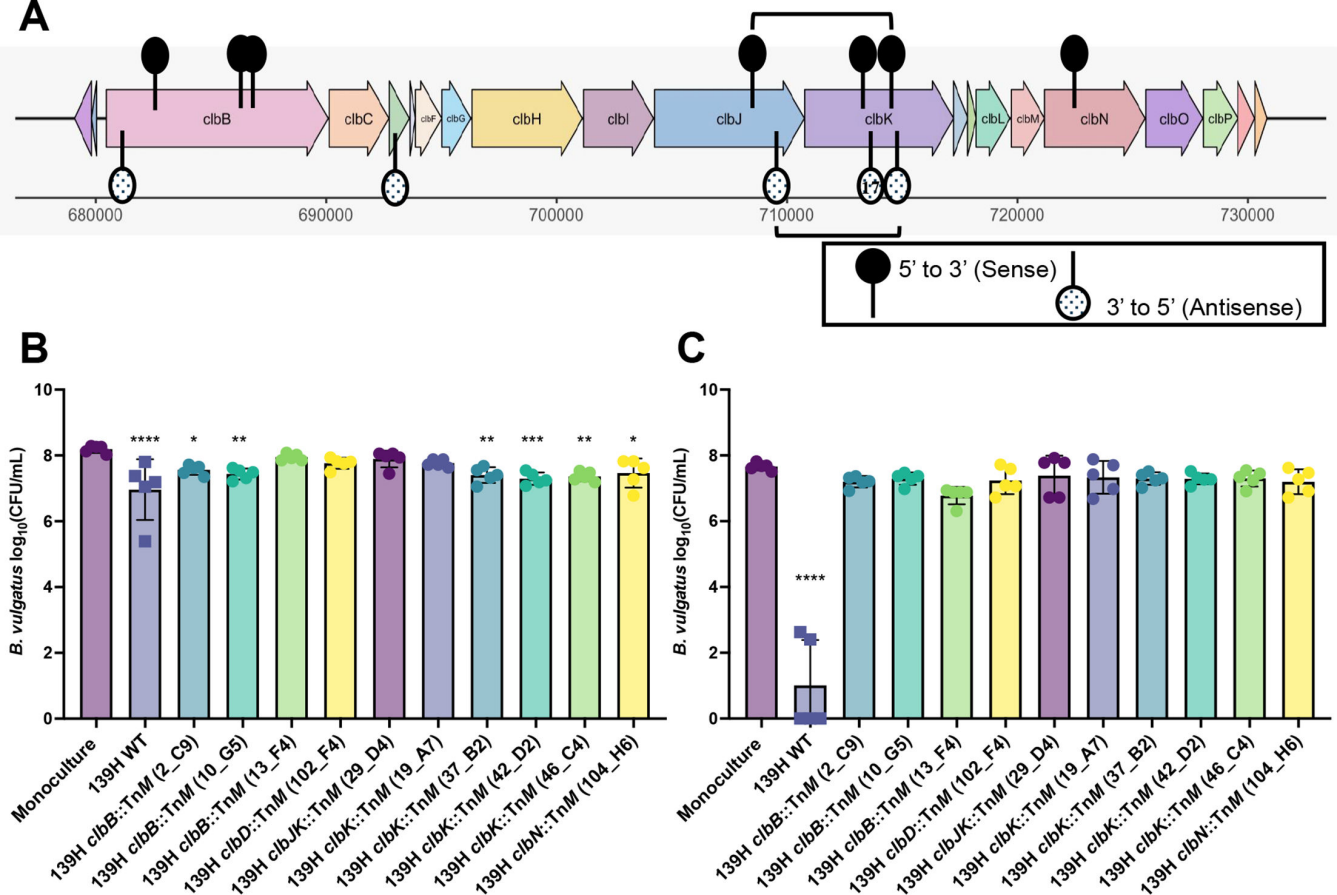
Transposon mutagenesis identified colibactin as necessary in *B. vulgatus* viability reduction by *E. coli* in the presence of glycerol. (**A**) Schematic of the *pks/clb* genetic island, spanning 54 kb and containing genes *clbA–clbS*. Black lollipops denote transposon insertions in the 5′–3′ direction, inverted white and black checkered lollipops denote transposon insertions in the 3′–5′ direction. There were 17 independent antisense hits to *clbK,* as represented by the highlighted “17.” Two independent candidates have transposon insertions detected in both *clbJ* and *clbK*, denoted with the bracket. (**B and C**) *B. vulgatus* clinical isolates (*n* = 5) grown in monoculture (first bar, purple) and co-culture with *E. coli* 139H WT (second bar, navy) and representative *E. coli clb*::Tn*M* mutants for 72 h in (**B**) MiPro pH 7 and (**C**) MiPro pH 7 + 1% glycerol. Statistical analysis performed using one-way ANOVA with Dunnett’s multiple comparisons between each co-culture condition with the monoculture control (*, *P* < 0.05; **, *P* < 0.01; ***, *P* < 0.005; ****, *P* < 0.001). Error bars represent mean +/− SEM.

The 26 independent transposon insertions spanned across five genes in the 54 kb *clb/pks* island, with single hits in *clbD* and *clbN*, and multiple hits in *clbB*, *clbJ,* and *clbK* (Fig. 3A; Table S11); 20 of the 26 transposon insertions were oriented in the 3′–5′ direction, or antisense, to the coding region. In addition to disrupting the function of the gene in which the transposon is inserted, the outward facing P*_tac_* promoter may be modulating the expression of the gene upstream of the insertion in these cases. In two independent candidates, the transposon was detected in both *clbJ* and *clbK*, denoted as “linked” with a bracket (Fig. 3A). In these strains, it appears that there were two transposon insertions: one in *clbJ* and one in *clbK*. Furthermore, two “partial restoration” candidates (*htpG*::Tn*M*) disrupted molecular chaperones that aid in colibactin synthesis (Table S10) (53), located outside the *pks* genetic island, further enforcing the role of colibactin synthesis in this phenotype.

We tested whether the mutants grew to the same extent as *E. coli* 139H WT. One representative mutant from each gene that was hit was cultured in monoculture for 72 h anaerobically in MiPro pH 7 and MiPro pH 7 + 1% glycerol. There were no observed growth advantages in glycerol-supplemented medium across all strains, including WT (Fig. S6A). However, one of the *clbJK*::Tn*M* “linked” candidates (29_D4) exhibited a slight but significant growth defect at the CFU/mL level compared to WT in MiPro + 1% glycerol. This observation may have contributed to *B. vulgatus* 127O’s ability not to show a reduction in co-culture with this mutant.

Next, we performed a tertiary validation screen with the representative *clb*::Tn*M* hits to confirm a full restoration of *B. vulgatus* 127O viability by determining CFU/mL for both microbes. In addition to *B. vulgatus* 127O, we tested four other strains of *B. vulgatus* to address whether this restoration of viability is applicable across strains. We co-cultured each *B. vulgatus* strain with *E. coli* 139H WT and with each *E. coli* Tn*M* mutant in MiPro pH 7 for 72 h and compared co-culture growth to the monoculture control (Fig. 3B). The WT strain significantly inhibited *B. vulgatus* strains in co-culture by ~1 log CFU/mL, while some of the transposon mutants showed restored *B. vulgatus* viability, with the exception of mutants *clbB*::Tn*M* (2_C9 and 10_G5), *clbK*::Tn*M* (37_B2 and 42_D2, 46_C4), and *clbN*::Tn*M* (104_H6). These transposon mutants significantly reduced *B. vulgatus* viability in co-culture, relative to monoculture controls, however, to a lesser degree than *E. coli* 139H WT. We repeated these co-culture assays in MiPro pH 7 + 1% glycerol and observed complete restoration of viability for all *B. vulgatus* strains across all tested transposon mutants (Fig. 3C). Thus, the restoration of viability of *B. vulgatus* was more consistent in glycerol (e.g., the conditions in which the mutant screen was performed) but was still observed in the absence of glycerol and translated across all tested *B. vulgatus* strains. Despite one transposon mutant (*clbJK*::Tn*M* 29_D4) with a slight but significant growth defect compared to WT, all tested *E. coli* 139H mutants grew similarly to WT in co-culture with *B. vulgatus* strains in both MiPro and MiPro +1% glycerol (Fig. S6B and C), suggesting that changes in endpoint *E. coli* CFU/mL are not responsible for driving *B. vulgatus* rescue.

Finally, we tested the growth kinetics of each representative transposon insertion against *E. coli* 139H WT to account for potential differences in log-phase growth. Here, we inoculated *E. coli* 139H WT and each representative transposon mutant in optically clear supplemented brain heart infusion broth (BHIS) buffered to pH 7 and BHIS pH 7 + 1% glycerol at OD_600_ = 0.01. Using a spectrophotometer in an anaerobic chamber at 37°C, we quantified OD_600_ of the strains in each condition every 3 min for 8 h. All strains grew similarly in BHIS pH 7, with no changes compared to the WT strain (Fig. S7A). In glycerol-supplemented medium, all transposon mutants, except for *clbK*::Tn*M* (37_B2), grew similarly to WT; the *clbK*::Tn*M* mutant had enhanced growth in late log-phase (2.9–3.4 h) compared to WT; however, the endpoint OD_600_ values between the two strains were similar (Fig. S7B). These results suggest that the restoration of *B. vulgatus* viability by these transposon mutants is independent of observed growth defects in the mutant strains compared to growth with *E. coli* 139H WT.

### Glycerol and microbial competition modulate colibactin production *in vitro*

We next sought to investigate the role of glycerol and microbial competition on colibactin production. Literature suggests that specific environmental and metabolic factors modulate expression of the *pks* island, including iron (54, 55), oligosaccharides (i.e., glucose and inulin) (56, 57), SCFAs (58, 59), oxygen (60), serine (61), spermidine, and inflammation (44). These *pks*-enhancing factors can be attenuated through the addition of inhibitory factors (i.e., ferrous sulfate in the case of iron [57]). To the best of our knowledge, glycerol has yet to be investigated as a potential modulator of colibactin regulation.

To test the hypothesis that glycerol coupled with microbial competition induces colibactin production, we performed ultra-performance liquid chromatography tandem mass spectrometry (UPLC–MS/MS) to quantify the prodrug scaffold that is hydrolyzed by the ClbP periplasmic peptidase during colibactin activation. The prodrug scaffold, *N*-myristoyl-d-Asn, is stable under aerobic conditions, unlike the final colibactin molecule (60), thus rendering it useful for quantification studies (62).

**Fig 4:**
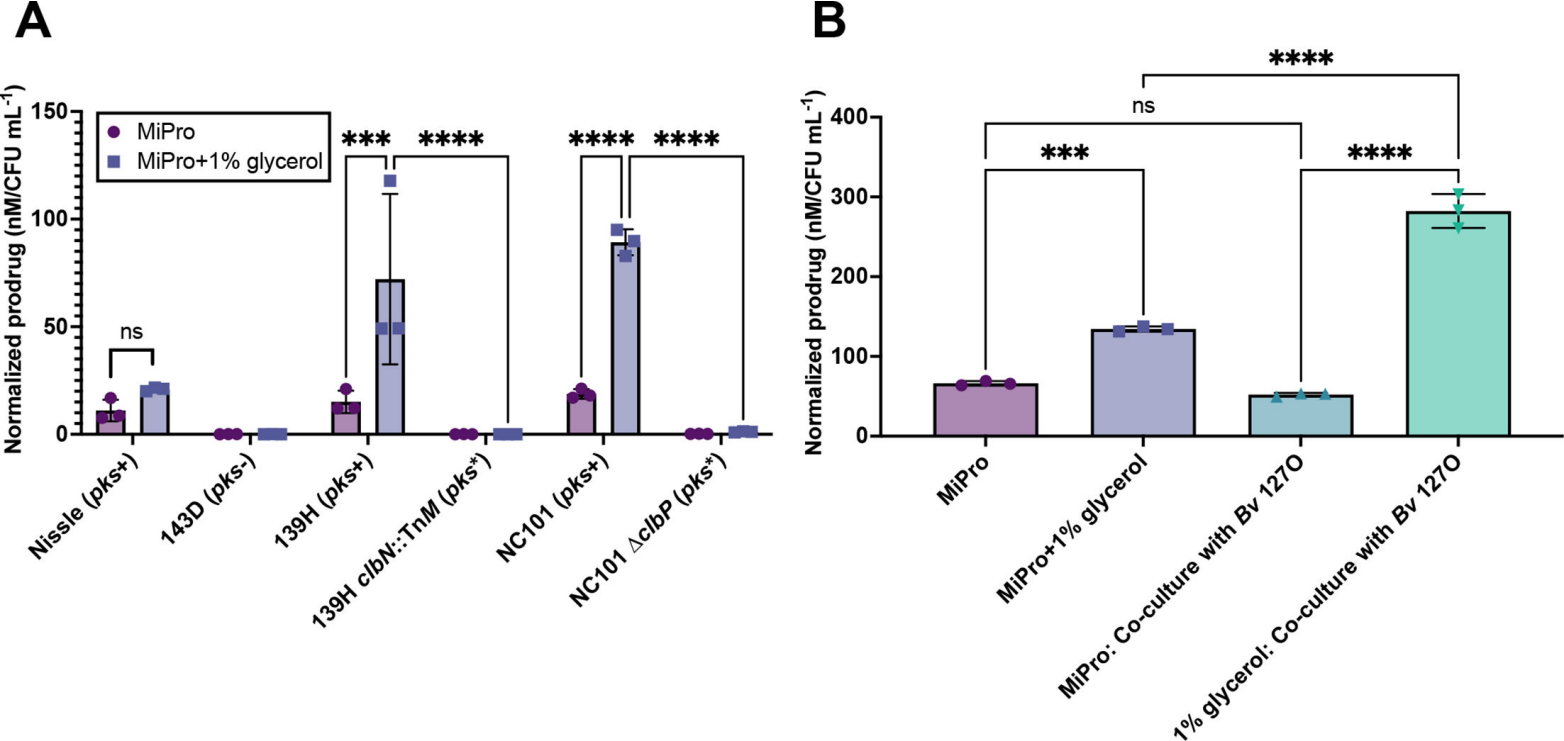
Glycerol and microbial competition modulate colibactin prodrug production *in vitro*. (**A**) *E. coli* strains (*n* = 6) were grown anaerobically in MiPro pH 7 (circles) and MiPro pH 7 + 1% glycerol (squares) for 24 h. Following incubation, cell count was quantified via CFU/mL plating on LB agar, and the levels of *N*-myristoyl-d-Asn, the colibactin-derived prodrug motif (labeled “prodrug”) were quantified in whole cultures using LC-MS. Prodrug concentrations were normalized to cell count (nM/CFU mL^−1^) across all conditions. Statistical analysis performed using two-way ANOVA with Šídák’s multiple comparisons (***, *P* < 0.005; ****, *P* < 0.001). (**B**) *E. coli* 139H was grown anaerobically in MiPro pH 7 and MiPro pH 7 + 1% glycerol with and without co-culture with *B. vulgatus* 127O for 72 h. Following incubation, cell count was quantified via CFU/mL plating on LB agar for *E. coli* and blood sheep agar + 100 µg/mL gentamicin for *B. vulgatus*. Levels of *N*-myristoyl-d-Asn, labeled “prodrug,” were quantified in whole cultures using LC-MS. Prodrug concentrations were normalized to *E. coli* cell count (nM/CFU mL^−1^) across all conditions. Statistical analysis performed using one-way ANOVA with Šídák’s multiple comparisons (***, *P* < 0.005; ****, *P* < 0.001; ns, not significant). Error bars represent mean +/− SEM.

First, we examined the difference in prodrug production across several *E. coli* strains: Nissle (*pks+*), 143D (*pks*−), 139H (*pks+*), 139H *clbN*::Tn*M* (*pks*-mutant*), NC101 (*pks+*), and NC101 ∆*clbP* (*pks*-mutant*). Monocultures of each isolate were grown anaerobically in MiPro and MiPro +1% glycerol for 24 h. Cell count was quantified via CFU/mL, and whole-culture prodrug concentrations were normalized to cell count. Overall, the *E. coli pks* + strains (e.g., Nissle, 139H, and NC101) produced moderate amounts of prodrug in MiPro (~11–18 nM/CFU mL^−1^; Fig. 4A), and this production was significantly increased in the presence of glycerol (~72–89 nM/CFU mL^−1^; Fig. 4A), with the exception of Nissle (21 nM/CFU mL^−1^). Notably, the *pks* mutants (e.g., 139H *clbN*::Tn*M* and NC101 ∆*clbP*) produced prodrug levels below the limit of detection, similar to the levels observed in the *pks-* strain 143D. Taken together, these results suggest that glycerol induces the production of colibactin (except in the Nissle strain), as quantified by measuring levels of *N*-myristoyl-d-Asn. Additionally, these findings confirm the elimination of colibactin production in both a *clbN* transposon mutant and a *clbP* null mutant.

Next, we investigated the role of microbial competition on colibactin production. *E. coli* 139H was grown in monoculture and co-culture with *B. vulgatus* 127O anaerobically in MiPro pH 7 and MiPro + 1% glycerol pH 7 for 72 h. Cell count was quantified via CFU/mL to confirm a reduction in *B. vulgatus* viability, and whole-culture prodrug concentrations were normalized to *E. coli* cell counts. Glycerol induced production of prodrug in 72 h cultures compared to MiPro alone (*P* < 0.0001; Fig. 4B). Co-culture with *B. vulgatus* in MiPro did not change prodrug concentrations relative to the MiPro monoculture control (*P* = 0.43). However, prodrug concentrations in co-cultures with glycerol were significantly increased compared to both the glycerol monoculture control (*P* < 0.0001) and MiPro co-culture (*P* < 0.0001; Fig. 4B), suggesting that both glycerol and microbial competition are required to maximize colibactin biosynthesis.

### *Pks+ E. coli* strains are found in both non-CF and CF infants and correlate with the depletion of *B. vulgatus*

We explored the prevalence of *pks* + strains of *E. coli* in the CF gut microbiome, hypothesizing that increased detection or isolation of colibactin-encoding strains of *E. coli* may alter microbial interactions in CF gut microbiomes. We characterized 22 *E. coli* isolates (CF: 6, non-CF: 15, lab: 1, originally isolated from healthy adult stool) from children’s stool or adult colonoscopy samples that have been whole genome sequenced. In the CF collection, 4/6 strains were isolated from children’s stool (*n* = 3 children), while 2/6 strains were isolated from a single adult CF colonoscopy sample (GIEHZ100). In the non-CF collection, all 15 strains were isolated from an adult non-CF colonoscopy sample (GIEHV103). The *E. coli* lab strain Nissle 1917, a recognized probiotic strain (63), contains the complete 19-gene *pks* island, including an annotated *clbS/dfsB* family gene that may or may not have a related or redundant function to the immunity protein *clbS*. We included this strain in this study, using its protein sequences as the reference in our comparative genomics study.

Using BLAST, we compared the Nissle Clb protein sequence to query the whole genome of each *E. coli* clinical isolate as the subject sequence. We determined which clinical isolates possessed homologous protein sequences to each Clb protein in the cluster, the percent identity, e-value, and other quality parameters compared to the Nissle reference, and where in the genome the island was located. We filtered the sequences by e-value (< 1E-10) and percent identity (> 50%); the clinical isolate sequences that did not meet this filtering threshold were marked as absent for that protein (Table S12). Next, we generated a heat map to display sequence similarity of the colibactin biosynthetic cluster across isolates (Fig. S8). The results from this comparative genomics analysis show that two CF (138G and 143D) and one non-CF (GP0236) stool *E. coli* isolates are devoid of any *clb* genes. One CF stool isolate (139G) and two CF colonoscopy isolates (GP0165 and GP0192) possess various genes encoding proteins in the cluster with relatively high sequence similarity to the Nissle sequence. However, these partial sequences all lack *clbA* and *clbR*, the two regulators of the cluster. The genes detected in GP0165 and GP0192 have 100% sequence similarity to each other, supporting the clonal nature of strains isolated longitudinally in the GI tract in a single person. Interestingly, only one CF isolate contains the full *pks* island (*E. coli* 139H). This strain and *E. coli* 139G were isolated from stool from the same child with CF within a 3-month span, suggesting acquisition or adaptation of colibactin production may arise in CF childhood. In the non-CF strain collection, 14/15 strains contain the full *pks* island. Only one strain, GP0236, lacks all proteins in the cluster. While most isolates from this donor appear clonal at the *pks* level, this result suggests that there may be populations of *E. coli* with various degrees of colibactin conservation within one GI tract.

**Fig 5:**
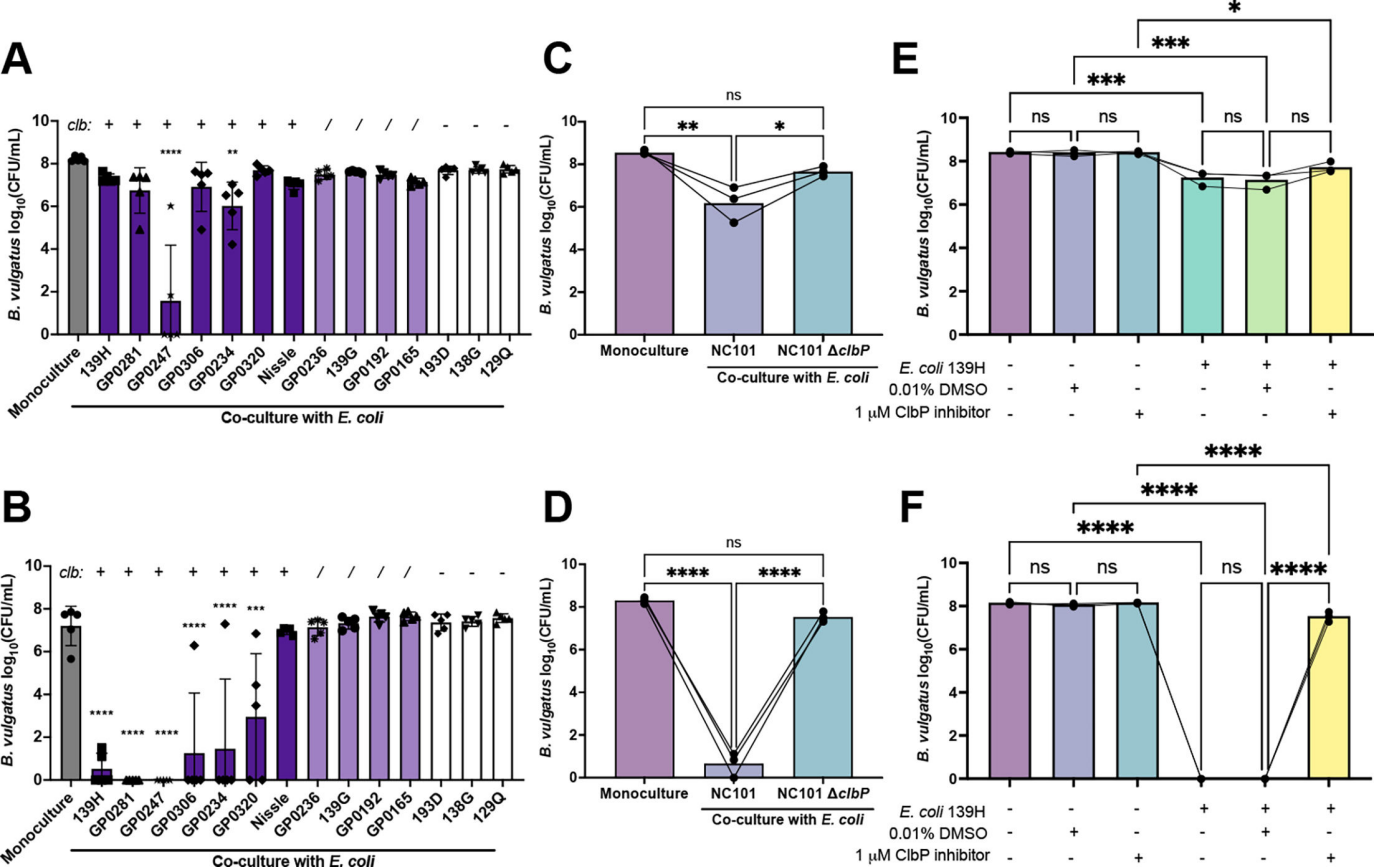
The *pks* gene cluster in *E. coli* correlates with reduced *B. vulgatus* viability in the presence of glycerol. (**A and B**) *B. vulgatus* strains (*n* = 5) grown anaerobically in monoculture (gray bar) and co-culture with 13 individual *E. coli* isolates in (**A**) MiPro and (**B**) MiPro + 1% glycerol for 72 h. *E. coli* strains with a complete *pks/clb* gene cluster are colored in dark purple (+), partial *pks/clb* gene cluster in light purple (/), and no *pks/clb* genes in black/white (−). Statistical analysis performed using one-way ANOVA with Dunnett’s multiple comparisons between co-culture CFU/mL to monoculture CFU/mL (*, *P* < 0.05; **, *P* < 0.01; ***, *P* < 0.005; ****, *P* < 0.001). (**C and D**) *B. vulgatus* strains (*n* = 3, CFPLTA003-2B, 127O, 139H) grown anaerobically in monoculture (first purple bar) and co-culture with *E. coli* NC101 (second light purple bar) and NC101 ∆*clbP* (third blue bar) in (**C**) MiPro and (**D**) MiPro + 1% glycerol for 72 h. The average log_10_(CFU/mL) of three biological replicates of each *B. vulgatus* strain is connected by a line. Statistical analysis was performed using one-way ANOVA with Tukey’s multiple comparisons (*, *P* < 0.05; **, *P* < 0.01; ****, *P* < 0.001). (**E and F**) *B. vulgatus* strains (*n* = 3, CFPLTA003-2B, 127O, 139H) grown anaerobically in monoculture (1st, 2nd, 3rd bars; “*E. coli* 139H –“) in (**E**) MiPro or (**F**) MiPro + 1% glycerol supplemented with 0.01% DMSO (2nd and 5th bars) or 1 µM ClbP inhibitor (3rd and 6th bars). The matrix below represents the presence (+) or absence (−) of co-culture with *E. coli* 139H, supplementation with 0.01% DMSO vehicle, or 1 µM ClbP inhibitor. The average log_10_(CFU/mL) of three biological replicates of each *B. vulgatus* strain is connected by a line. Statistical analysis was performed using one-way ANOVA with Tukey’s multiple comparisons (*, *P* < 0.05; ***, *P* < 0.005; ****, *P* < 0.001; ns, not significant).

These results reveal that colibactin-producing strains of *E. coli* can be isolated from gut microbiome samples of both CF and non-CF genotypes, although our patient sample size is small (CF: 5, non-CF: 1). The age demographics of the genotype cohorts are also different, where the CF cohort is 80% children, and the non-CF cohort is 100% adult. Additionally, we isolated these strains via culture-dependent methods, which may introduce biases. Because there is evidence that *E. coli* may acquire the colibactin biosynthetic cluster via horizontal gene transfer (64), age, and *E. coli* abundance likely play a role in detection. Thus, to better examine the role of colibactin on microbial competition, we performed *in vitro* competition assays with *B. vulgatus* and various *E. coli* clinical isolates, hypothesizing that *pks + E. coli* strains would reduce *B. vulgatus* viability more than strains lacking these genes. The results from the co-culture assays grown in MiPro suggest that the viability of *B. vulgatus* strains is significantly reduced by some *E. coli* strains that possess the full suite (+) of *clb* genes (*E. coli* GP0247 and GP0234; Fig. 5A). Other *E. coli* strains that contain only some of the *clb* genes (/) or none of the *clb* genes (−) do not reduce *B. vulgatus* viability when co-cultured in MiPro. In MiPro + 1% glycerol, all *E. coli clb +* strains significantly reduce *B. vulgatus* viability, relative to its monoculture growth (Fig. 5B). In contrast, there is no effect by *E. coli clb*(/) or *clb*(−) strains. This observation is independent of *E. coli* growth and isolate source (Fig. S9A and B). The one exception to this observed phenotype is *E. coli* Nissle, which is *clb+*. This strain does not inhibit *B. vulgatus* under either growth condition. This observation is likely explained by the reduced concentration of prodrug produced by Nissle in glycerol-supplemented medium (Fig. 4A). In addition, Nissle may produce metabolites, substrates, or other factors that support *B. vulgatus* growth in co-culture and ameliorate microbial competition with resident microbes. This phenomenon has been explored in numerous studies which generally support the use of Nissle as a probiotic strain, despite its *pks* + status (65, 66).

Next, we aimed to test the necessity of colibactin production in the observed interaction with *B. vulgatus*. To do this, we applied both genetic and chemical approaches. First, we obtained an *E. coli* strain lacking *clbP*, which encodes the periplasmic peptidase responsible for hydrolyzing the prodrug and activating colibactin. We co-cultured three independent *B. vulgatus* strains with the *clbP* mutant and its parent strain, NC101, in MiPro pH 7 (Fig. 5C) and MiPro pH 7 + 1% glycerol (Fig. 5D). The results from these co-cultures suggest that NC101, a *pks* + strain of *E. coli*, reduces viability in all tested isolates of *B. vulgatus* in MiPro, and this reduction is exacerbated in glycerol-supplemented medium. Furthermore, when *clbP* is absent, this reduction in viability is rescued, similar to monoculture controls, in both growth conditions. *E. coli* cell counts remain largely unchanged compared to its monoculture growth control (Fig. S9C and D), supporting the lack of association with *E. coli* growth.

We also tested the role of colibactin through small molecule inhibition of ClbP (62). This previously reported inhibitor is derived from a boronate ester precursor, (*S*)-*N*-(3-amino-3-oxo-1-(4,4,5,5-tetramethyl-1,3,2-dioxaborolan-2-yl)propyl)−4-phenylbutanamide, which spontaneously hydrolyzes in water to yield the active boronic acid inhibitor, (*S*)-(3-amino-3-oxo-1-(4-phenylbutanamido)propyl)boronic acid. This compound binds with high potency and selectivity to ClbP and mimics the hydrogen-bonding interactions of precolibactin, thus disrupting the hydrolysis of this biosynthetic intermediate (62). When *E. coli* 139H is incubated with 1 µM of the ClbP inhibitor for 72 h, prodrug concentrations are significantly reduced both in the presence and absence of glycerol, compared to the DMSO vehicle control (Fig. S10), confirming the potency of the compound. Furthermore, when *E. coli* 139H is grown in anaerobic co-culture with three independent *B. vulgatus* strains in MiPro pH 7 supplemented with either DMSO vehicle control or 1 µM of the ClbP inhibitor for 72 h, there is a modest yet nonsignificant rescue of *B. vulgatus* viability in the presence of the inhibitor (Fig. 5E). Importantly, in co-cultures with 1% glycerol, the ClbP inhibitor significantly restores *B. vulgatus* viability nearly to monoculture levels (Fig. 5F). Again, this restoration in viability is independent of changes in *E. coli* growth (Fig. S9E and F). Taken together, these findings support the sufficiency of colibactin production in the reduction in *B. vulgatus* viability in our system.

### Analysis of CF stool metagenomes suggests increasing trends in prevalence and abundance of *clb* genes

To address the discrepancy in cohort demographics from which the *E. coli* strains were isolated (e.g., difference in ages and sample type between CF and non-CF cohort), we investigated the prevalence and abundance of colibactin-related genes in stool samples from cwCF and those without. This analysis aimed to eliminate culture biases and focus on one age demographic: children under 3 years old. We utilized publicly available stool metagenomic data of CF and non-CF children (6), extracted DNA from CF and non-CF stool samples (*n* = 18 for each genotype) and performed metagenomic sequencing. In all, our data set includes 208 CF and 2,560 non-CF stool metagenomes. Human reads were filtered, microbial taxonomy was assigned, and the differential relative abundance of each taxon was calculated between genotypes of children (CF vs. non-CF). Microbial gene families and pathways were assigned using the UniRef90 database. Statistical analyses were performed using Wilcoxon rank-sum test to account for genotype.

**Fig 6:**
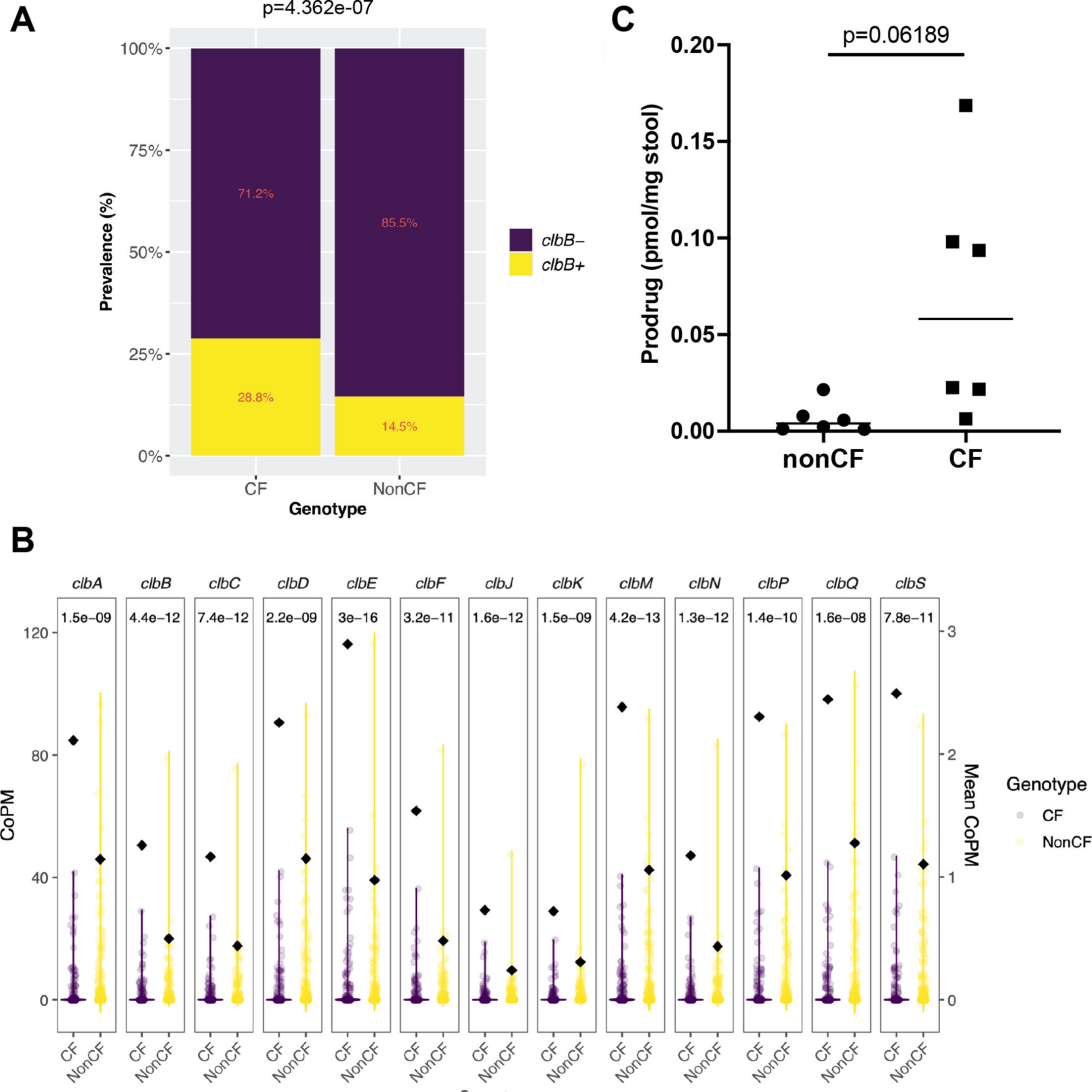
Markers of increased colibactin production are evident in CF stool. (**A**) Prevalence (%) of stool metagenomes with detectable *clbB* gene sequences (yellow, *clbB+*) or not (purple, *clbB*–) in CF and non-CF children. Statistical analysis was performed using the χ² test with Yates continuity correction, with a supplemental Fisher’s exact test. (**B**) Abundance for 12/19 gene families in the *clb* island, not assigned to a particular bacterial species (“unclassified”), are first normalized for gene length with reads per kilobase and then normalized for library depth, as depicted by copies per million (CoPM). Each colored point represents the CoPM of a given gene in one CF (purple) or non-CF (yellow) sample (left y-axis). Mean gene counts within each genotype are displayed for stool metagenomes as represented through the black diamonds (right y-axis). Statistical analysis performed using Wilcox rank-sum test to account for genotype; *P*-values are displayed at the top of each gene facet. (**C**) The colibactin prodrug was quantified from raw stool collected from non-CF (*n* = 6) and CF children (*n* = 6). Concentrations were normalized to the weight of each stool sample (pmol/mg stool). Statistical analysis performed using mixed-effect linear modeling with batch as the random variable and genotype as the fixed variable.

We first were interested in the prevalence of detectable *clb* genes in stool metagenomes. We focused on *clbB* due to its representation among transposon candidates and conservation in *pks+ E. coli* clinical isolates. We observed that 372/2,560 (14.5%) of non-CF stool metagenomes had detectable *clbB* levels, compared to 60/208 (28.8%) of CF stool metagenomes (*P* = 4.362e–07; Fig. 6A), indicating that colibactin-related genes are 2.38 times more prevalent in stool from cwCF. To our knowledge, this is the first study to identify an association between colibactin production and the CF genotype (26, 27). Because our analysis is focused on children under the age of 3 years, these results point to an enrichment of colibactin-related processes in younger CF cohorts than previously examined.

Next, we quantified the gene abundance of *clb* genes in CF and non-CF stool metagenomes. Gene abundance values indicate the frequency of a gene in a population; of note, frequency does not necessarily equate to gene expression or function, since coding variants that are nonfunctional may contribute to gene count. Our approach provides insight into the potential functional capabilities of colibactin production in these two microbiomes. Gene abundance for all tested genes*,* as displayed as mean copies per million (CoPM) reads (67), is significantly higher in CF stool metagenomes compared to non-CF (Fig. 6B). Because there is also an enrichment of *E. coli* in the CF gut, we plotted *clbB* gene abundance against the relative abundance of *E. coli* and found a significant correlation (Pearson R^2^ = 0.44, *P* = 0; Fig. S11A). Taken together, these results suggest that CF gut microbiomes possess higher levels of colibactin-associated genes, which are directly correlated with *E. coli* relative abundance.

Other species of Enterobacteriaceae, like *Klebsiella pneumoniae,* can synthesize colibactin and contribute to gene counts in our data set; however, the abundance of *clbB* gene counts did not correlate with *K. pneumoniae* relative abundance in CF stool metagenomes (Pearson R^2^ = −0.04, *P* = 0.545; Fig. S11B). Furthermore, because of our *in vitro* observations, we examined the correlation between *clbB* gene counts and *B. vulgatus* relative abundance. As with *Klebsiella* relative abundance*, clbB* gene counts did not correlate with levels of *B. vulgatus* in CF stool metagenomes (Pearson R^2^ = 0.01, *P* = 0.835; Fig. S11C).

In non-CF stool metagenomes, *clbB* significantly correlated with *E. coli* (Pearson R^2^ = 0.26, *P* = 0; Fig. S11D), although to a lesser extent than that observed in CF stool metagenomes. These results may suggest a contribution by other colibactin-producing bacteria; however, no correlation was found between *clbB* and *K. pneumoniae* (Pearson R^2^ = 0, *P* = 0.897; Fig. S11E). Thus, these findings likely reflect differences in *E. coli* relative abundance between genotypes and may suggest an enhanced role for *E. coli-*mediated colibactin production in CF and its potential as a biomarker in this disease cohort. Interestingly, there was a weak yet significant anticorrelation between *clbB* gene counts and *B. vulgatus* in non-CF stool metagenomes (Pearson R^2^ = −0.06, *P* = 0.03; Fig. S11F), supporting the *in vitro* observation that *E. coli pks*+ strain reduce *B. vulgatus* viability under baseline conditions lacking increased glycerol.

Finally, we investigated the concentration of *N-*myristoyl-d*-*Asn, the colibactin prodrug motif, in CF and non-CF stool samples (*n* = 6 each genotype). We homogenized raw stool, performed a solvent-based extraction, and quantified the prodrug motif using UPLC–MS/MS, analogous to quantification of the prodrug in *E. coli* cultures. The concentration was normalized to stool weight (nM/mg stool). Statistical analysis was performed using a mixed effect linear model to account for genotype (fixed variable) and batch effects (random variable). We observed a non-significant trend in increased colibactin prodrug in CF stool compared to non-CF stool (*P* = 0.062; Fig. 6C). Taken together, these results indicate an enrichment of colibactin-encoding genes and a potentially significant increase in the colibactin prodrug in CF stool compared to non-CF stool, although a larger study is needed to determine if this is a consistent finding. Given colibactin’s association with inflammation and CRC development, quantifying these markers in stool may aid in the early detection of CRC risk factors in young CF cohorts.

## DISCUSSION

In this study, we report that *Bacteroides*, specifically *B. vulgatus,* is sensitive to both CF-relevant *in vitro* growth conditions and microbial competition with *E. coli.* Under baseline conditions in MiPro, a medium used to mimic the environment of the healthy intestine (28), *B. vulgatus* strains show reduced viability starting at 8 h when in co-culture with *E. coli*. The competition between *E. coli* and *B. vulgatus* is exacerbated in certain CF-like conditions attributed to nutritional malabsorption, particularly high bile and high glycerol concentrations. To date, the role of glycerol in CF gut microbial interactions has not been explored. Through probing the role of glycerol in the competition model, we determined that the reduction in viability depends on live cells; cell-free supernatants of *E. coli* and *E. coli-B. vulgatus* co-cultures grown in glycerol do not recapitulate this reduction. This finding may indicate the role of transient and local acute mediators or direct antagonism.

We chose to take a genetic approach to identify *E. coli* genetic factors involved in this interaction. We generated a mariner transposon library in the CF-isolated *E. coli* strain 139H and performed a co-culture screen with a susceptible *B. vulgatus* strain (127O) in the presence of glycerol. Results from this screen highlighted the role of metabolism and nutrient competition in the microbial interaction, as well as definitively pointed to the biosynthesis of colibactin, a genotoxin associated with increased risk of CRC. *E. coli* mutants with transposon insertions in the *clb/pks* gene cluster rescue *B. vulgatus* growth in glycerol-supplemented co-cultures, independent of changes in *E. coli* growth. The involvement of colibactin production was further validated with a *clbP* null mutant of *E. coli* and a chemical inhibitor of ClbP, both of which restored *B. vulgatus* viability in co-culture with glycerol.

In this study, the significant impact of *E. coli* on *B. vulgatus* viability was striking, given the prior stool-based studies in a similar pediatric CF cohort (6). Here, CF gut microbiome samples cluster separately from non-CF counterparts by beta diversity measurements, driven largely by relative abundances of *B. bifidum*, *B. vulgatus,* and *E. coli*. These results identify key species driving CF dysbiosis in this age group, suggesting their potential as microbial biomarkers. The data also highlight the importance of microbial interactions and changes in species levels in driving compositional shifts. Using a CF-relevant *in vitro* system, we support a putative mechanism for the observed clustering, extending our findings to an additional eight clinical isolates from three *Bacteroides* species.

Several mechanisms may explain the colibactin-mediated competition between *pks+ E. coli* and *B. vulgatus*. First, evidence suggests that variations in microbial composition are attributed to colibactin production (68, 69). Mouse microbiomes with *pks+ E. coli* cluster separately from those without, driven by a decrease in Firmicutes (69). Functional analyses indicate enhanced DNA repair pathways, suggesting the role of genotoxic activity on resident bacteria (69). Additional studies show that *pks+ E. coli* induces DNA damage in distant microbes in a contact-independent (70) and oxygen-dependent manner (68). Notably, cells up to 100 µM away from colibactin-producing *E. coli* grown on soft agar plates exhibit a DNA-damage response within several hours of exposure (70). Second, the *clb/pks* island encodes colibactin and other intermediates and metabolites produced by NRPS-PKS (42, 44). The *pks* island is regulated by low iron levels, with expression induced by the ferric iron uptake regulator Fur binding to the promoter region of *clbA* (69). When active, the pathogenicity island also influences siderophore and microcin production, enhancing the microbe’s iron-scavenging capabilities and colonization (44). Siderophore production is crucial for competitive advantage against pathogens, such as *Salmonella* Typhimurium (65, 71). In addition, the *pks* pathway produces lipopeptides like C12-Asn-GABA, an analgesic that may contribute to the probiotic activity of *pks* + Nissle by reducing inflammation and promoting gut barrier activity, and C14-Asn, which has weak antimicrobial activity against *Bacillus subtilis* strains (42). Through the extensive process of colibactin production, there is a large diversity of intermediate metabolites (e.g., precolibactins) that are produced, some of which at high abundances with undetermined bioactivity (72). These metabolites likely contribute to interspecies competition in the intestine, although the environment that dictates their regulation and biological functions remains unclear. Third, *pks + E. coli* may antagonize other microbes, including *B. vulgatus*, by inducing prophage.

This process, dependent on colibactin production, activates lytic replication and kills bacterial cells, potentiating a phage epidemic in the broader microbial community (73). *pks+ E. coli* induces prophage across genetically dissimilar bacteria, including *Salmonella, Staphylococcus, Citrobacter, Enterococcus,* and *pks*– strains of *E. coli* (74). Thus, this observation raises the possibility that *pks*+ strains of *E. coli* kill *B. vulgatus* strains harboring resident prophage in our system, a finding that may have a wider array of ecological implications *in vivo*.

The role of glycerol in this interaction is unresolved yet suggests a connection between the *pks* machinery and fatty acid metabolism (72). Some of the most abundant alternative metabolites produced by the *pks* machinery contain malonyl-CoA moieties (72, 75, 76), suggesting crosstalk between colibactin and primary fatty acid biosynthesis. Considering this link, the enrichment of glycerol in the CF intestine may, in part, feed the production of *pks*-driven alternative metabolites. Further research is needed to determine the correlation between free glycerol levels and the regulation of the *pks* pathway, colibactin, and alternative metabolite production.

Several limitations should be considered when interpreting these findings. First, all experiments were conducted in liquid medium under *in vitro* conditions, which do not fully recapitulate the gut environment’s physical and nutritional complexity. Second, only four bacterial species were evaluated, precluding conclusions about community-wide responses or competitive interactions within a diverse microbiome. It is key to note that we do observe the same shifts in these key taxa when we examine complex stool populations (28). Third, glycerol was tested over a limited concentration range, likely higher than physiological levels, and other dietary fats or carbon sources were not examined. It is important to note that we observed *E. coli*-mediated reduction in *Bacteroides* even in low levels of glycerol in MiPro medium. This study lacks dietary intake metadata for the pediatric cohort from which we obtained stool; meal timing likely impacts the glycerol availability in the gut. Importantly, while the prodrug quantification results point to the trending increase in colibactin in CF stool, the absolute concentration of colibactin in the gut is unknown due to its extreme instability and recalcitrance to isolation (59). Inference of colibactin activity via prodrug quantification and metagenomics analyses may shed light on its potential for production under certain physiologies; however, these approaches do not confirm enzymatic activity and genotoxicity *in vivo*. Colibactin’s instability in microaerophilic environments suggests that its activity in the gut environment is restricted to anoxic regions, and thus, the toxin likely acts locally—this reasoning may explain the lack of toxicity by supernatants on *B. vulgatus* isolates. Therefore, bulk analysis of stool may miss the intricacies of colibactin activity in terms of *in vivo* microbial competition.

Microbial imbalances in the developing CF intestine have significant clinical implications. Longitudinal studies show that gut microbiome alterations in CF, such as reduced diversity measures, depletion of beneficial taxa, and expansion of pro-inflammatory taxa, precede and predict clinical measures (32, 77). Breastfed infants and those with higher Shannon Diversity Index scores in stool have a longer time to first pulmonary exacerbation (77), suggesting a protective role of a diverse gut microbiome for respiratory health. Additionally, reduced abundances of key commensal taxa, including *Parabacteroides,* in CF cohorts before initial lung colonization with *P. aeruginosa* further support the gut-lung axis in this disease context (77). Recent research highlights *Bacteroides-*mediated SCFAs, specifically propionate, as a key modulator of lung and systemic inflammatory cytokine levels (78). Importantly, propionate production was similar across 20 clinical isolates of various *Bacteroides* species, highlighting the universal anti-inflammatory nature of the genus.

Numerous species produce SCFAs in the GI tract, but many are significantly reduced in CF cohorts compared to controls, leading to lower overall SCFA concentrations (8). Depletion of a single taxon, like *B. vulgatus*, may not lead to detectable reductions in SCFA abundances or drive clinical inflammation. However, the loss of one species in an ecosystem can reduce others through metabolic cross-feeding, colonization resistance, and direct antagonism (79). Evidence suggests that *B. vulgatus* partially drives compositional differences in CF versus non-CF gut microbiomes (6). Thus, the *in vivo* ecological consequences of colibactin-mediated depletion of *B. vulgatus* likely extend to other clinically relevant commensal microbes. Future studies are needed to determine whether these responses reflect broader gut microbial metabolism features.

Here, we show that colibactin from *pks + E. coli* significantly reduces the viability of *B. vulgatus* isolates, particularly in high glycerol, a reduction that can be reversed with genetic and chemical inhibitors. We also found increased *clb* gene counts and colibactin prodrug levels in CF stool samples compared to non-CF counterparts. Given the correlation between *E. coli* abundance and *clb* gene counts, coupled with the feasibility of sampling stool at young ages, we argue that screening for *pks + E. coli* in infants with CF may provide insight into the risk of development of microbial dysbiosis and later inflammatory conditions, including CRC. Because fat malabsorption is not exclusive to CF populations, colibactin-mediated microbial dysbiosis may be relevant to other diseases, including Crohn’s and ulcerative colitis, and highlight a potential focus for prognostics and therapeutics across disease cohorts.

## MATERIALS AND METHODS

Detailed descriptions of strains, media, growth conditions, co-culture assays, linear regression, glycerol quantification, supernatant assays, mariner transposon mutagenesis, quantification of prodrug in bacterial cultures and stool, ClbP inhibitor assays, *E. coli* DNA extraction and whole genome sequencing, homology analysis, stool DNA isolation and sequencing, metagenomic sequencing analyses, and all associated statistical analyses are provided in the supplemental material.

## SUPPLEMENTAL MATERIAL

Supplemental material jb.00190-26-s0001.pdf Supplemental materials, Figures S1 to S11, and Tables S1 to S10.

Table S2 jb.00190-26-s0002.xlsx Summarized growth data.

Table S11 jb.00190-26-s0003.xlsx Mutants mapped to the clb island.

Table S12 jb.00190-26-s0004.xlsx Filtered blastp results of Clb proteins.

ASM does not own the copyrights to Supplemental Material that may be linked to, or accessed through, an article. The authors have granted ASM a non-exclusive, world-wide license to publish the Supplemental Material files. Please contact the corresponding author directly for reuse.
